# Peripheral vasoconstriction influences thenar oxygen saturation as measured by near-infrared spectroscopy

**DOI:** 10.1007/s00134-012-2486-3

**Published:** 2012-02-14

**Authors:** Alexandre Lima, Michel Egide van Genderen, Eva Klijn, Jan Bakker, Jasper van Bommel

**Affiliations:** Department of Intensive Care Adults, Erasmus MC University Medical Centre Rotterdam, P.O. Box 2040, 3000 CA Rotterdam, The Netherlands

**Keywords:** Near-infrared spectroscopy, Skin temperature, Body surface cooling, Capillary refill time

## Abstract

**Purpose:**

Near-infrared spectroscopy has been used as a noninvasive monitoring tool for tissue oxygen saturation (StO_2_) in acutely ill patients. This study aimed to investigate whether local vasoconstriction induced by body surface cooling significantly influences thenar StO_2_ as measured by InSpectra model 650.

**Methods:**

Eight healthy individuals (age 26 ± 6 years) participated in the study. Using a cooling blanket, we aimed to cool the entire body surface to induce vasoconstriction in the skin without any changes in central temperature. Thenar StO_2_ was noninvasively measured during a 3-min vascular occlusion test using InSpectra model 650 with a 15-mm probe. Measurements were analyzed for resting StO_2_ values, rate of StO_2_ desaturation (RdecStO_2_, %/min), and rate of StO_2_ recovery (RincStO_2_, %/s) before, during, and after skin cooling. Measurements also included heart rate (HR), mean arterial pressure (MAP), cardiac output (CO), stroke volume (SV), capillary refill time (CRT), forearm-to-fingertip skin-temperature gradient (Tskin-diff), perfusion index (PI), and tissue hemoglobin index (THI).

**Results:**

In all subjects MAP, CO, SV, and core temperature did not change during the procedure. Skin cooling resulted in a significant decrease in StO_2_ from 82% (80–87) to 72% (70–77) (*P* < 0.05) and in RincStO_2_ from 3.0%/s (2.8–3.3) to 1.7%/s (1.1–2.0) (*P* < 0.05). Similar changes in CRT, Tskin-diff, and PI were also observed: from 2.5 s (2.0–3.0) to 8.5 s (7.2–11.0) (*P* < 0.05), from 1.0°C (−1.6–1.8) to 3.1°C (1.8–4.3) (*P* < 0.05), and from 10.0% (9.1–11.7) to 2.5% (2.0–3.8), respectively. The THI values did not change significantly.

**Conclusion:**

Peripheral vasoconstriction due to body surface cooling could significantly influence noninvasive measurements of thenar StO_2_ using InSpectra model 650 with 15-mm probe spacing.

## Introduction

Near-infrared spectroscopy (NIRS) is a noninvasive technique that allows the determination of tissue oxygenation based on spectrophotometric quantitation of oxy- and deoxyhemoglobin levels within a tissue. Since its advent as a noninvasive monitoring tool for peripheral tissue oxygenation, a relationship between the potential influences of skin circulation on tissue oxygen saturation (StO_2_) signals has been debated. Numerous studies have investigated different NIRS devices in various tissues and under various experimental conditions [1–4]. These studies indicate that the StO_2_ signal is widely influenced by the volume of the vascular bed in the catchment area of the probe. Current clinical NIRS studies, particularly those performed in an intensive care setting seem to neglect this relationship when monitoring peripheral tissue oxygenation. One of the reasons may be the lack of studies that address the influence of thenar skin circulation on StO_2_ signal as measured with the most current commercial device available (InSpectra model 650).

We recently reported in an observational study that abnormalities in skin perfusion contribute significantly to the StO_2_-derived signal measured with an InSpectra model 650 probe on the thenar, and this correlation was independent of disease condition or systemic hemodynamics [5]. The question that remains is how important is the potential contribution of low skin blood flow, specifically of the thenar eminence, on StO_2_? To answer this question, we performed StO_2_-derived tissue oxygenation measurements during local vasoconstriction induced by extremity cooling. We hypothesize that the decrease in skin blood flow resulting from peripheral vasoconstriction during body surface cooling significantly influences the noninvasive measurement of thenar StO_2_ using an InSpectra model 650 with 15-mm probe spacing.

## Materials and methods

### Study population

The study was conducted at a university-affiliated teaching hospital. We recruited healthy volunteers with no history of receiving any vasoactive medication. The volunteers were instructed to avoid caffeine-containing drinks for 24 h before the experiments. The local medical ethics committee approved this study protocol.

### Measurements

#### StO_2_-derived tissue oxygenation

StO_2_-derived tissue oxygenation was continuously monitored using an InSpectra tissue spectrometer model 650 with a 15-mm probe over the thenar eminence. A vascular occlusion test (VOT) was performed by arrest of forearm blood flow using a conventional sphygmomanometer pneumatic cuff. The cuff was placed around the upper arm and was inflated to a pressure approximately 30 mmHg greater than patient systolic pressure for 3 min. On the completion of the ischemic period, the occluding cuff was rapidly deflated to 0 mmHg. VOT-derived StO_2_ parameters were divided into three components: resting StO_2_ values, rate of StO_2_ desaturation (RdecStO_2_, expressed as %/min), and rate of StO_2_ recovery (RincStO_2_, expressed as %/s).

#### Peripheral perfusion

Peripheral perfusion was evaluated using conventional physical examination with capillary refill time (CRT), forearm-to-fingertip skin-temperature gradient (Tskin-diff), perfusion index (PI), and tissue hemoglobin index (THI). CRT was measured by applying firm pressure to the distal phalanx of the index finger for 15 s. A chronometer recorded the time for the return of the normal color. The Tskin-diff was obtained with two skin probes (Hewlett Packard 21078A) attached to the index finger and on the radial side of the forearm, midway between the elbow and the wrist. Tskin-diff can better reflect changes in cutaneous blood flow than skin temperature itself. When being evaluated under constant environmental conditions, Tskin-diff increases during vasoconstriction, and a threshold of 4°C has been shown to reflect vasoconstriction in critically ill patients [6]. The PI provides a noninvasive method for evaluating perfusion and has been shown to reflect changes in peripheral perfusion [7]. In this study, the PI value was obtained using Masimo pulse oximetry, which displays a range from 0.02% (very weak pulse strength) to 20% (very strong pulse strength). The THI was derived from a second-derivative attenuation spectrum and is part of the StO_2_ algorithm of the NIRS monitor.

#### Global hemodynamic parameters

Global hemodynamic parameters included heart rate (HR), stroke volume (SV), cardiac output (CO), and mean arterial pressure (MAP). Global parameters were recorded using thoracic bioimpedance, as measured by a noninvasive cardiac output monitor (NICOM; Cheetah Medical Inc., Wilmington, DE, USA). The NICOM system and technology have been described elsewhere [8]. In summary, connecting the NICOM to the subject requires four double electrode stickers placed on the thorax, according to the manufacturer’s instructions. Data are automatically recorded using a computer data logger on a minute-by-minute basis.

#### Cooling techniques and monitoring

Body surface cooling was achieved using circulating cold water blankets (Thermowrap, Or-Akiva Ind. Park, Israel). A cooling pump device (CSZ Blanketrol III, model 233, Cincinnati SubZero, Inc.) was connected to the blankets to pump cold water. The water temperature was set to the desired temperature. The blanket garment was attached directly to the patient’s body using medically approved adhesive.

### Protocol

Individuals were positioned in supine position dressed with the cooling blankets on a comfortable bed. The blanket suit covered the entire body with the exception of the head, instrumented forearm, hands, and feet. The cooling pump device permitted control of blanket water temperature by changing the temperature of water perfusing the suit. The suit was then perfused with 32°C water. Electronic measurements were obtained continuously and the values are reported as averaged data for each interval. The time points included the following: baseline measurements prior to the cooling process (*T*_0_), after 30 min of peripheral cooling (*T*_1_), and after 30 min of the suspension of cooling and initiation of the rewarming process (*T*_2_). For this protocol, peripheral cooling was designed mainly to chill the skin over the entire body to induce only skin vasoconstriction without any changes in central temperature. Therefore, core temperature was measured each 5 min with an infrared tympanic thermometer (First Temp Genius model 3000A). Ambient temperature was constant during all experiments (*T* = 22°C). Skin vasoconstriction was defined as a minimal 50% decrease either in the Tskin-diff temperature or the PI signal.

### Statistical analysis

The results are presented as the median (25th–75th), unless otherwise specified. A one-way repeated-measures ANOVA was conducted to compare NIRS-derived and peripheral perfusion parameters prior, during, and after rewarming. The Bonferroni post hoc test was performed if a significant main effect was observed. SPSS (version 15.0, SPSS, Chicago, IL) was used for statistical analysis. A *P* value less than 0.05 was considered statistically significant.

## Results

Eight healthy individuals (4 male, 4 female) participated in the study. The mean age, height, and weight were 26 ± 6 years, 172 ± 5 cm, and 74.1 ± 6.2 kg, respectively. Table 1 lists the global hemodynamic variables stratified by the time points of the study. All subjects tolerated the cooling process well and did not develop shivering during the experiment. We found a nonsignificant tendency towards an increase in SV and CO during peripheral cooling at *T*_1_ (Table 1). Core temperature and heart rate did not change significantly during the experiment.

**Table 1:** Descriptive analysis of global hemodynamic variables and central temperature stratified by the time points

	Time point		
	*T*_0_	*T*_1_	*T*_2_
Heart rate (bpm)	76 (70;83)	67 (58;73)	70 (67;74)
Stroke volume (ml)	110 (76;125)	139 (132;147)	114 (106;164)
Cardiac output (l/min)	8.6 (8.0;9.4)	10.1 (8.9;12.2)	7.9 (7.3;10.1)
Mean arterial pressure (mmHg)	93 (88;101)	100 (92;107)	95 (87;100)
Central temperature (°C)	36.8 (36.6;36.9)	36.6 (36.4;36.9)	36.6 (36.4;36.9)

Data presented as the median (25th;75th)

*T*_*0*_ prior to the cooling process, *T*_*1*_ after 30 min of peripheral cooling, *T*_*2*_ after 30 min of the suspension of cooling and initiation of the rewarming process

**Fig. 1:**
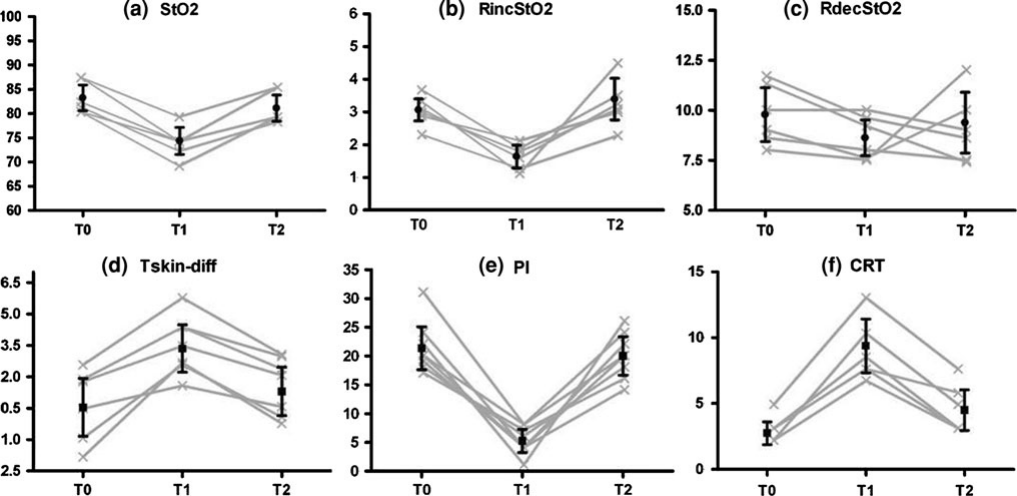
Time course of NIRS-derived variables and peripheral perfusion parameters. *T*_0_, prior to the cooling process; *T*_1_, after 30 min of peripheral cooling; *T*_2_, after 30 min of the suspension of cooling and initiation of the rewarming process. **a** StO_2_, peripheral tissue oxygenation (%); **b** RincStO_2_, rate of StO_2_ recovery after arterial occlusion (%/s); **c** RdecStO_2_, rate of StO_2_ desaturation during arterial occlusion (%/min); **d** Tskin-diff, forearm-to-fingertip skin-temperature gradient (°C); **e** PI, perfusion index (%); **f** CRT, capillary refill time (s). *Lines* represent individual values for each healthy volunteer. *Bars* are mean ± 95% CI

Figure 1 presents the time course of the NIRS dynamic variables and peripheral perfusion parameters before, during, and after the skin cooling process. Table 2 lists the absolute values of all peripheral parameters as stratified by time points. The peripheral cooling resulted in skin vasoconstriction in all volunteers with a significant decrease in the Tskin-diff temperature and in the PI signal: 55% (40–175) and 77% (62–83) compared with baseline, respectively. Concomitantly, we observed a significant decrease in StO_2_ and RincStO_2_ values but not in RdecStO_2_. The rewarming process increased StO_2_ and RincStO_2_ values towards baseline levels. Similar changes in CRT, Tskin-diff, and PI were also observed. The THI values did not change significantly during the entire experiment.

**Table 2:** Descriptive analysis of NIRS-derived variables and peripheral perfusion parameters stratified by the time points

	Time point		
	*T*_0_	*T*_1_	*T*_2_
StO_2_ (%)	82 (80;87)	72* (70;77)	80 (79;85)
RincStO_2_ (%/s)	3.0 (2.8;3.3)	1.7* (1.1;2.0)	3.2 (3.0;4.2)
RdecStO_2_ (%/min)	9.5 (8.0;11.6)	8.6 (7.5;9.6)	8.8 (7.7;11.5)
THI (a.u.)	13.1 (12.8;13.9)	13.2 (12.6;13.9)	13.3 (12.9;14.2)
Tskin-diff (°C)	1.0 (−1.6;1.8)	3.1* (1.8;4.3)	1.2 (−0.3;2.7)
CRT (s)	2.5 (2.0;3.0)	8.5* (7.2;11.0)	4.0 (3.0;5.7)
PI (%)	10.0 (9.1;11.7)	2.5* (2.0;3.8)	9.1 (8.2;11.7)

Data presented as the median (25th;75th)

*T*_*0*_ prior to the cooling process, *T*_*1*_ after 30 min of peripheral cooling, *T*_*2*_ after 30 min of the suspension of cooling and initiation of the rewarming process, *RincStO*_*2*_ rate of StO_2_ increase after arterial occlusion, *RdecStO*_*2*_ rate of StO_2_ deoxygenation during arterial occlusion, *THI* tissue hemoglobin index, *Tskin*-*diff* forearm-to-fingertip skin-temperature gradient, *CRT* capillary refill time, *PI* perfusion Index

* *P* < 0.05 versus *T*_*0*_ and *T*_*2*_ (one-way repeated-measures ANOVA with Bonferroni post hoc test)

We were also interested in investigating which of the NIRS variables was most affected by changes in skin circulation. Our results indicated that the magnitude of changes seem to be more prominent in RincStO_2_ than StO_2_ and that RincStO_2_ is more sensitive to changes in peripheral perfusion than StO_2_ itself. When compared with baseline values, the magnitude of the RincStO_2_ decreases was larger than that observed for StO_2_ [47% (30–62%) vs. 11% (9.2–13.1), *P* = 0.001].

## Discussion

The key finding from this study is that changes in vasomotor tone in the skin of the thenar eminence contributed significantly to the StO_2_-derived parameters as measured with a NIRS InSpectra device. The main mechanistic theory of our study is that peripheral vasoconstriction due to surface cooling results in decreased perfusion of the skin and, therefore, in parallel, changes in the StO_2_ resting values and in the StO_2_ recovery rate. Under resting conditions, the impact of peripheral perfusion alterations on NIRS-derived measurements can be expected to be magnified as the skin temperature decreases.

This finding was not totally unexpected because light from the NIRS system must pass through the skin and some absorbance in the resistance vessels that supply subepidermal capillaries would be anticipated. The 15-mm NIRS probe mainly covers approximately an 8-mm depth of tissue and focusing on the muscle. Skin and subcutaneous layers above the muscle definitely contribute to the overall StO_2_ measured. It is likely that the decreasing StO_2_ effect after skin cooling is mainly due to the upper layers’ compromised perfusion because of cutaneous vasoconstriction. One may argue that it is possible that the cooling device induced changes beyond that of skin circulation and that flow in skeletal muscle was also altered. Our study does not allow us to conclude which of the two components (skin or muscle) is the major contributor to the changes in StO_2_-derived variables in our model. Nevertheless, we speculate that participation of muscle blood flow was not predominant because THI readings in our volunteers presented small changes during the cooling period. On the other hand, participation of skin blood flow was significant, as reflected by changes in skin temperature, PI, and CRT. The THI represents the total tissue concentration of hemoglobin in both extravascular and vascular tissue, and its physiological significance and clinical utility are still under investigation. Our model induced changes mainly on the arterial side of microcirculation and may explain why THI was not affected by the peripheral cooling device, as the sensitivity of THI is greater for vessels with high capacitance, such as post capillaries and venous compartments. This phenomenon may explain why other NIRS researchers have reported low THI values and normal StO_2_ in patients with sepsis, as this is a condition related to vascular leak and low vascular density due to microcirculatory derangements [9, 10]. Therefore, the decreased NIRS signal from oxygenated hemoglobin is likely the result of a decrease in arterial blood volume within the peripheral vasculature as a consequence of vasoconstriction.

We chose this model to test our hypotheses because previous studies, including one by our own group, have shown that peripheral vasoconstriction is a frequent abnormality in critically ill patients [11, 12]. For instance, studies that employed NIRS as a peripheral tissue oxygenation monitoring device have shown that the fall in StO_2_ in peripheral tissues correlates well with the degree of hypotension in trauma and hemorrhagic shock [13–16]. However, these findings were always related to acute shock states and the disturbance of the systemic circulation, which indicates that the pathological link between hypotension and the fall in StO_2_ may be explained by increased peripheral vasoconstriction as a result of the adrenergic response that follows the neurohumoral compensatory mechanisms induced by the low-flow systemic shock state. Peripheral vasoconstriction may very well explain the fall in StO_2_ levels in acute situations, such as in trauma and cardiogenic or hemorrhagic shock. In critically ill patients after resuscitation of the systemic circulation, during the stability phase, peripheral vascular tone may no longer reflect the acute compensatory mechanisms because others factors overcome this physiologic response; such factors include mechanical ventilation, vasopressors, vasodilators, sedatives, and opiate use. However, abnormalities in peripheral perfusion may persist despite patient systemic hemodynamic stability [11, 12, 17]. The noticeable decreases in StO_2_ and StO_2_ recovery rate in our model provide evidence that peripheral vasoconstriction markedly influences the NIRS measurements of thenar tissue oxygenation and may confound interpretation of StO_2_-derived parameters in critically ill patients, in whom peripheral perfusion constantly changes over time.

Another interesting finding was that we could induce significant changes in StO_2_-derived tissue oxygenation values by maintaining unchanged systemic hemodynamics. We found a nonsignificant tendency towards an increase in SV and CO during peripheral cooling. This finding may be explained by the shift of blood volume from the vasoconstricted peripheral circulation to the central circulation with a subsequent increase in the cardiac preload, justifying the augmentation of cardiac output as a result of an increase in stroke volume. In one previous study conducted by our group, we found that changes in StO_2_-derived parameters were correlated with parameters of peripheral perfusion in critically ill patients but were independent of the hemodynamic status of the patient [5]. In another recent study in a model of controlled central hypovolemia, a decreased venous return with a concomitant decrease in stroke volume did not lead to clinically significant changes in StO_2_ as measured on the thenar [18]. These findings strongly suggest that StO_2_-derived parameters are more affected by changes in peripheral vasomotor tone than by systemic hemodynamic conditions. Perhaps even more interesting to the clinician who applies NIRS for peripheral perfusion monitoring is the knowledge that abnormal StO_2_-derived parameters may reflect a condition of peripheral vasoconstriction independent of systemic hemodynamics.

In conclusion, the presence of peripheral vasoconstriction due to body surface cooling could significantly influence the noninvasive measurement of thenar StO_2_ using an InSpectra model 650 with 15-mm probe spacing. Depending on the condition of peripheral circulation, significant decreases in peripheral blood flow can affect StO_2_-derived measurements, particularly StO_2_ and StO_2_ recovery rate, which are exclusively dependent on local vasodilation capacity. Therefore, careful consideration must be given when using NIRS to measure tissue oxygenation in critically ill patients, and consideration should be given to the peripheral circulation when interpreting peripheral tissue oxygenation.
